# Association of markers of inflammation on attention and neurobehavioral outcomes in survivors of childhood acute lymphoblastic leukemia

**DOI:** 10.3389/fonc.2023.1117096

**Published:** 2023-06-21

**Authors:** Yin Ting Cheung, Kenneth Kin-Wah To, Rong Hua, Chui Ping Lee, Agnes Sui-Ying Chan, Chi Kong Li

**Affiliations:** ^1^ School of Pharmacy, Faculty of Medicine, The Chinese University of Hong Kong, Hong Kong, Hong Kong SAR, China; ^2^ Neuropsychology Laboratory, Department of Psychology, The Chinese University of Hong Kong, Hong Kong, Hong Kong SAR, China; ^3^ Department of Paediatrics, Faculty of Medicine, The Chinese University of Hong Kong, Hong Kong, Hong Kong SAR, China; ^4^ Department of Paediatrics and Adolescent Medicine, Hong Kong Children’s Hospital, Hong Kong, Hong Kong SAR, China; ^5^ Hong Kong Hub of Paediatric Excellence, The Chinese University of Hong Kong, Hong Kong, Hong Kong SAR, China

**Keywords:** pediatric leukemia, survivors, cognitive function, inflammation, late effects, psychooncology, biomarkers

## Abstract

**Background:**

Survivors of childhood acute lymphoblastic leukemia (ALL) are at-risk of developing cognitive impairment and neurobehavioral symptoms. Inflammation induced by a compromised health status during cancer survivorship is proposed as a pathophysiological mechanism underlying cognitive impairment in cancer survivors.

**Objectives:**

To evaluate the associations of biomarkers of inflammation with attention and neurobehavioral outcomes in survivors of childhood ALL, and to identify clinical factors associated with biomarkers of inflammation in this cohort.

**Methods:**

We recruited patients who were diagnosed with ALL at ≤ 18 years old and were currently ≥5 years post-cancer diagnosis. The study outcomes were attention (Conners Continuous Performance Test) and self-reported behavioral symptoms (Adult Self-Report [ASR] checklist). Using a commercial screening kit, survivors’ plasma (5ml) was assayed for 17 cytokines/chemokine cell-signaling molecules that are associated with neurodegenerative diseases. The final panel of the targeted markers included interleukin (IL)-8, IL-13, interferon-gamma (IFN*-*γ), monocyte chemoattractant protein*-*1 *(*MCP*-*1), macrophage inflammatory protein-1β, and tumor necrosis factor-*α*. Biomarker levels were rank-ordered into tertiles based on the sample distribution. Multivariable general linear modeling was used to test for associations between biomarkers and study outcomes in the overall cohort and stratified by gender.

**Results:**

This study included 102 survivors (55.9% males, mean[SD] age 26.2[5.9] years; 19.3[7.1] years post-diagnosis). Survivors within top tertiles of IFN-γ (Estimate =6.74, SE=2.26; *P*=0.0037) and IL-13 (Estimate =5.10, SE=2.27; *P*=0.027) demonstrated more inattentiveness. Adjusting for age, gender and treatment, more self-reported thought (Estimate=3.53, SE=1.78; *P*=0.050) and internalizing problems (Estimate =6.52, SE=2.91; *P*=0.027) correlated with higher IL-8. Higher levels of IL-13 (RR = 4.58, 95% CI: 1.01–11.10) and TNF-α (RR = 1.44, 95% CI: 1.03–4.07) were observed in survivors had developed chronic health conditions (n=26, 25.5%). The stratified analysis showed that association of IFN-γ with attention was stronger in male survivors than in female survivors.

**Conclusion:**

Inflammation due to cancer-related late effects may potentially be mechanistic mediators of neurobehavioral problems in pediatric ALL survivors. Markers of inflammation can potentially be applied to assess or monitor the effectiveness of interventions, particularly behavioral interventions, in improving cognitive outcomes in survivors. Future work includes understanding the underlying gender-specific pathophysiology behind functional outcomes in the population.

## 1 Introduction

The overall survival rate of childhood acute lymphoblastic leukemia (ALL) is approaching 90% in most developed regions and countries (1–4). This remarkable success is attributed to more precise assessment diagnostic strategies and contemporary treatment protocols for pediatric ALL (2, 4). While improving the survival rates of children with cancer in resource-limited settings requires continual effort, attention is now targeted at the emerging population of survivors. In particular, many studies have observed that survivors of childhood ALL develop poorer functional outcomes that manifest as cognitive and behavioral deficits (5, 6). Survivors demonstrated inattentiveness and poorer sustained attention during the post-treatment phase (7, 8). In addition, survivors may also report behavioral symptoms such as inattention, sluggish cognitive tempo and psychological problems (9, 10). It is well documented that central nervous system (CNS)-directed treatments, such as intrathecal chemotherapy, cranial radiation therapy (CRT), and high-dose methotrexate (HDMTX), can directly affect attention and behavioral outcomes in survivors (6, 11). Survivors may also develop chronic health conditions (CHCs) that can indirectly affect long-term CNS outcomes. For example, pulmonary, endocrine and cardiovascular complications may exert additional stress and damage on the brain, leading to cognitive and behavioral deficits in survivors (11–13).

Radiation treatment and many chemotherapy drug exert their anticancer effects through inducing inflammation-induced reactive oxygen or nitrogen species (14). Recent studies have proposed that cancer treatment may damage the CNS indirectly through the production of free radicals and inflammatory biomarkers in cancer survivors (6, 15, 16). The therapy-elicited inflammation subsequently illicit the development of cognitive and behavioral symptoms in cancer patients. For example, one study identified elevated uric acid, a marker of stress and vascular injury, as being associated with poor executive function and processing speed in adolescent survivors of childhood ALL (17). Poorer cognitive flexibility and visual–motor processing speed were observed in survivors of childhood leukemia with higher circulating levels of inflammatory markers (17). These collective findings raise the possibility that the cancer or the cancer treatment experience may have initiated a subclinical process of injury and stress in key organ systems that may affect their brain function, gradually evolving as clinically evident cognitive and neurobehavioral impairments as the survivors advance into adulthood. However, few studies have evaluated the association among chronic health status (i.e. the development of CHCs), inflammation and cognitive/behavioral outcomes in survivors.

**Figure 1:**
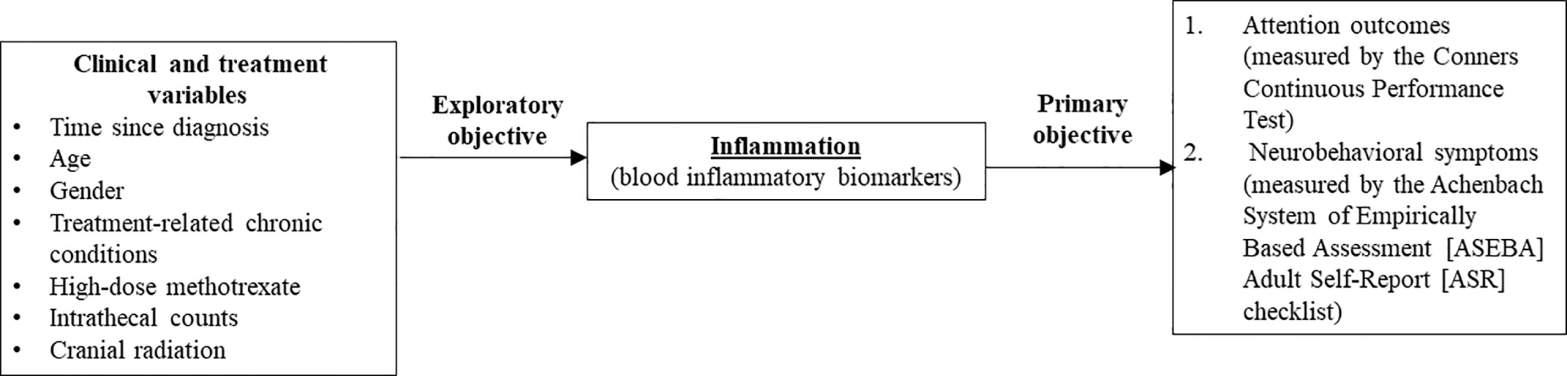
Theoretical framework. This figure summarizes the theoretical framework and objectives of this study. Dashed arrow: Studies within the field of pediatric oncology survivorship literature have identified that demographic and clinical risk factors (11–13, 17–19) are associated with attention and neurobehavioral outcomes in survivors of childhood acute lymphoblastic leukemia. Primary objective – This study hypothesized that systemic inflammation (as reflected by higher blood cytokine levels) is associated with poorer attention and neurobehavioral outcomes. Exploratory objective – To identify the association between specific demographic and clinical risk factors with inflammation markers in survivors.

The primary objective of this study was to evaluate the associations of biomarkers of inflammation with functional outcomes (attention and neurobehavioral symptoms) in survivors of childhood ALL in Hong Kong. The exploratory objective was to examine the relationship between clinical risk factors and inflammation in this cohort. Our study hypotheses are presented in **Figure 1**. We hypothesize that survivors with higher blood cytokine levels would demonstrate poorer attention and neurobehavioral outcomes than survivors with lower cytokine levels, and certain clinical risk factors would be associated with elevated inflammatory status, as reflected by higher cytokine levels.

## 2 Methods

### 2.1 Study design

This was a cross-sectional study conducted at the Survivors’ Clinic of the Prince of Wales Hospital in Hong Kong. They study period was between June 2018 and June 2020. Prior to the commencement of this study, approval was obtained from the Joint Chinese University of Hong Kong – New Territories East Cluster Clinical Research Ethics Committee (Ref: 2017.701). Written informed consent was obtained from all participants.

### 2.2 Study population

Survivors of childhood ALL were eligible for inclusion in the study if they were diagnosed with ALL at ≤ 18 years old by a pediatric oncologist, ≥ 18 years of age, and ≥ 5 years post-cancer diagnosis and ≥ 2 years post-cancer treatment at the time of follow-up assessment. Survivors were excluded if had a history of cancer relapse, developed secondary cancers, had any genetic or pre-existing (i.e. diagnosed before cancer diagnosis) condition associated with cognitive impairment (e.g., Down syndrome, developmental disorder such as autism spectrum disorder, attention deficit hyperactivity disorder) or significant head injuries, or were pregnant or lactating.

All patients received standard treatment protocols that included combinations of intravenous HDMTX with leucovorin rescue, intrathecal chemotherapy, oral prednisone and dexamethasone, anthracyclines, l-asparaginase, cytarabine, and cyclophosphamide (20). Based on the clinical presentations of leukemia at diagnosis and evaluation of minimal residual disease, the patients were stratified into standard-risk, intermediate-risk, or high-risk protocols. Based on clinical definition, intravenous HDMTX is defined as a single-dose of more than 1 g/m^2^ of methotrexate with leucovorin rescue. Children in the low-risk arm received HDMTX at 2 gm/m^2^ per dose for four cycles (total 8 gm/m^2^) and 13 to 18 triple intrathecal treatments (MTX, hydrocortisone, and cytarabine), in addition to other chemotherapeutic agents. Children in the standard-/high-risk arm received HDMTX at 5.0 gm/m2 per dose for four doses (total 20 gm/m^2^) and 16 to 25 triple intrathecal treatments. A quarter of the survivors (n = 28, 27.4%) who were treated in the late 1990s/early 2000s received cranial radiation (total 1,800 cCy in 10 fractions).

### 2.3 Study outcomes

For this study, the functional outcomes referred to attention and neurobehavioral outcomes. Attention was the cognitive domain of interest as attention deficits have been observed among survivors of ALL in the literature (7, 8, 21, 22). Survivors’ attention outcomes were evaluated using the Conners Continuous Performance Test-III (CPT-III) (23), a commonly used computerized test in other international studies (8, 22, 24). The attention measures of interest were attentiveness (CPT omissions, variability, and detectability), impulsivity (CPT commissions and perseveration), and sustained attention (standard deviation and inter-stimulus interval of the CPT hit reaction time) (23). The CPT-III is a widely used tool to diagnose attention deficits in the local population (25, 26), and it has previously been utilized in cognitive studies among childhood cancer survivors (27, 28) and the general pediatric/adolescent population in Hong Kong (29, 30).

Neurobehavioral functioning was evaluated using the traditional Chinese version of the Achenbach System of Empirically Based Assessment (ASEBA) Adult Self-Report (ASR) checklist (31). The primary outcomes of interest were the syndrome scales, which referred to attention problems, thought problems, internalizing problems (comprising somatic complaints, anxiety/depressive symptoms and withdrawn behavior), externalizing problems (comprising aggressive behavior, intrusive behavior, and rule-breaking behavior), and sluggish cognitive tempo. The Chinese version of the ASR has been used and validated in the Chinese general population (32).

### 2.4 Biological markers

On the day of testing, 5 mL of blood was drawn from each participant and placed in ethylenediaminetetraacetic acid tubes. The collected blood samples were centrifuged at 1140* g* (2500 rpm) for 30* min* and stored according to standard procedures until assay at the Core Laboratory of the Li Ka Shing Institute of Health Sciences, the Chinese University of Hong Kong. The resulting plasma samples (50 μL each) were assayed in triplicate using a highly sensitive multiplex immunoassay (Luminex^®^). During analysis, a solution of 50 μL diluted beads per well was used. A coefficient of variation of less than 20% for the triplicate assessments was considered acceptable. In addition, extrapolation of the plasma cytokine concentrations below the lowest limit of quantitation was calculated using the Bio-Plex Manager™ software.

We selected a commercially available screening panel that is previously utilized in studies on biomarkers clinically related to neurodegenerative diseases (33–35). The Bio-Plex Pro Human Cytokine Screening Panel Catalog Number M5000031YV) included interleukin (IL)-1β, IL-2, IL-4, IL-5, IL-6, IL-7, IL-8, IL-10, IL-12, IL-13, IL-17A, granulocyte colony-stimulating factor (G-CSF), granulocyte-macrophage colony-stimulating factor (GM-CSF), interferon gamma (IFN-γ), monocyte chemoattractant protein-1 (MCP-1), macrophage inflammatory protein-1β (MIP-1β), and tumor necrosis factor-α (TNF-α).

The assay was conducted in two stages. In the first screening stage, 25 samples served as pilot samples for identifying the following cytokine and chemokine cell-signaling molecules present in the plasma at concentrations above the detection limits. While 25 samples might not be adequate to identify any trends/patterns in the levels of cytokines, it was reasonable to assume that 25 samples in the initial screening step might still provide valid information on identifying cytokines that are below detection levels in a research setting. It was decided *a priori* that markers that were below the detectable limit in ≥85% of the pilot samples were excluded from subsequent analyses; accordingly, 1β, IL-2, IL-4, IL-5, IL-6, IL-7, IL-10, IL-12, G-CSF, and GM-CSF were excluded due to low detection levels.

In the second stage of the analysis, the remaining samples were assayed using a customized Bio-Plex Pro™ human cytokine immunoassay kit. The final panel of the targeted markers contained IL-8, IL-13, IFN-γ, MCP-1, MIP-1β, and TNF-α. The reported associations of these biomarkers with cognitive and neurobehavioral function in the literature (17–19, 36–53) are presented in **Supplement 1**.

At the data analysis stage, the biomarker levels were rank-ordered into tertiles based on the sample distribution of the cohort. We did not dichotomize survivors based on the age-based thresholds as the reference ranges for cytokines varied widely depending on different clinical conditions and measurement methods (54, 55). Furthermore, the thresholds used for the diagnosis of clinical conditions might not apply when evaluating the effects of cytokines on functional outcomes such as cognitive function. This approach has been adopted by other cognitive studies involving biomarkers (17, 22, 56, 57).

### 2.5 Clinical and treatment variables

To identify clinical risk factors associated with inflammatory markers in the exploratory objective, the survivors’ clinical information (age at diagnosis and CHCs) and treatment-related variables (types and cumulative doses of chemotherapy drugs, CRT), were abstracted from the electronic health data repository. Consistent with other studies in childhood cancer survivors (12, 13, 58, 59), treatment-related CHCs were defined as health conditions that were diagnosed during or after the completion of cancer treatment. Conditions that existed before cancer diagnosis and treatment were not considered CHCs. For this study, we only considered cardiopulmonary (ICD-9 codes 390–459), endocrine/metabolic (240–279), and neurological (320–359) health conditions as previous studies have reported associations between these conditions with cognitive and neurobehavioral outcomes in survivors of cancer (11–13, 17, 58, 59).

### 2.6 Statistical analysis

In this study, the sample characteristics and outcome measures were described using descriptive statistics. All of the neurocognitive and neurobehavioral measures were scaled into age-adjusted T-scores (mean = 50; standard deviation [SD] = 10) according to published normative data. A higher T-score was indicative of more severe impairment or problems. A one-sample *t* test was used to compare the survivors’ performance with population norms (T-score = 50). Only measures on which the survivors differed from the normative samples at P < 0.05 (after Bonferroni correction) were included in subsequent analyses.

Multivariable general linear modeling (GLM) was used to examine the associations of CHCs and inflammatory markers with attention/neurobehavioral outcomes. The basic model included age at follow-up (years), age at diagnosis (years), gender, and treatment factors (CRT and HDMTX). Subsequently, the variables of interest (markers of inflammation [top tertile/other tertiles]) were added to the basic model.

As emerging evidence suggests sex-specific presentation of functional outcomes in survivors of childhood cancer (17, 21, 22, 59, 60), GLM was stratified by biological sex as a sensitivity analysis. The strength of the associations was presented as unstandardized estimates (Est) and standard errors (SE).

Finally, the exploratory analysis included multivariable log-binomial models (i.e., GLMs with Poisson error and log-link function) to examine the associations of clinical factors (gender, age, time since diagnosis, and CHCs) and treatment factors (CRT, the number of intrathecal injections, and HDMTX [8 versus 20 g/m^2^]) with markers of inflammation. The corresponding relative risk (RR) estimates and 95% confidence intervals (CIs) were calculated.

All analyses were conducted using SAS ^®^ OnDemand for Academics (SAS Institute Inc). *P* < 0.05 was considered statistically significant, and all statistical tests were two-sided.

## 3 Results

Of the 160 survivors screened for eligibility, 126 survivors provided consent and completed neurocognitive assessments. After excluding survivors who did not provide blood samples (n = 12), survivors who had unmeasurable specimens (e.g., hemolyzed samples; n = 6) and survivors with missing treatment information (n = 6), the data of 102 survivors were analyzed (**Supplement 2**).

### 3.1 Clinical and treatment characteristics

The final sample consisted of data on 102 survivors (mean age = 26.2, SD = 5.9 years) (**Table 1**). On average, they were 19.3 (SD = 7.1; range [8.0 – 29.5]) years post-cancer diagnosis, and 14.5 (SD = 7.0, range [5.6 – 25.4]) years from the completion of cancer treatment. The survivors had received standard-risk (n = 32, 31.3%), intermediate-risk (n = 42, 41.2%), or high-risk (n = 28, 27.5%) protocols. A minority received CRT (n = 28, 27.4%), while the others were treated with chemotherapy-only protocols (n = 74, 72.6%). One-fourth of the survivors (n = 26, 25.5%) developed at least one CHC, including cardiopulmonary (n = 10, 9.8%), endocrine/metabolic (n = 8, 7.8%), and neurological (n = 5, 5.9%) disorders (**Supplement 3**).

**Table 1:** Demographics and clinical characteristics (n = 102).

Characteristics	No. (%)	Mean (SD)
Demographics and Clinical		
Gender		
Male	57 (55.9)	
Female	45 (44.1)	
Highest education (years)		
Secondary school and below	36 (35.3)	
Post-secondary and above	66 (64.7)	
Age at diagnosis (years)		6.8 (4.7)
Age at evaluation (years)		26.2 (5.9)
Time since diagnosis (years)		19.3 (7.1)
Time since completion of treatment		14.5 (7.0)
Risk stratification		
Low risk	32 (31.3)	
Standard risk	42 (41.2)	
High risk	28 (27.5)	
Treatment modality		
Cranial radiation	28 (27.4)	
Chemotherapy-only protocol	74 (72.6)	
Chemotherapy		
IV high-dose methotrexate* (g/m^2^)		15.7 (7.2)
8g/m^2^	29 (28.4)	
20 g/m^2^	73 (71.6)	
Intrathecal chemotherapy (no. of injections)		15.6 (4.2)
Chronic health conditions		
Any	26 (25.5)	
Cardiopulmonary	10 (9.8)	
Endocrine/metabolic	8 (7.8)	
Neurology	5 (4.9)	

IV, intravenous; SD, standard deviation.

*Total high-dose methotrexate was also categorized as 8 g/m^2^ (4 cycles of 5 g/m^2^) of methotrexate versus 20 g/m^2^ (4 cycles of 5 g/m^2^).

### 3.2 Attention and neurobehavioral outcomes

Survivors performed worse than the normative sample on measures of sustained attention (CPT hit reaction time SD mean [SD] 56.2 [6.4]), inattentiveness (CPT omissions 54.5 [2.6] and CPT detectability 55.1 [9.1]), and impulsivity (CPT commissions 54.4 [7.7]) (all *P* = 0.0008, corrected for false discovery rate) (**Table 2**). The rates of attention impairment ranged from 2.9% to 12.8%.

**Table 2:** Attention and neurobehavioral outcomes.

		Attention outcomes			
		Mean (SD) *T*-Scores*	Impaired %^	95% CI	*P*#
**CPT HRT ISI change (sustained attention)**		51.0 (7.6)	2.9	0.0 – 6.0	1.0
**CPT HRT SD (sustained attention)**		56.2 (6.4)	7.8	2.6 – 13.0	**0.0008**
**CPT Variability (inattentiveness)**		52.1 (9.7)	2.9	0.0 – 6.2	0.23
**CPT Omissions (inattentiveness)**		54.5 (2.6)	7.8	2.6 – 13.0	**0.0008**
**CPT Detectability (inattentiveness)**		55.1 (9.1)	12.8	6.2 – 19.2	**0.0008**
**CPT Perseverations (impulsivity)**		51.6 (7.1)	2.9	0.0 – 6.0	0.18
**CPT Commissions (impulsivity)**		54.4 (7.7)	4.9	0.7 – 9.0	**0.0008**
		Behavioral outcomes (Syndrome scales)			
		Mean (SD) *T*-Scores*	Impaired %^	95% CI	*P*#
**Attention problems**		55.6 (7.9)	13.7	7.0 – 20.4	**0.0006**
**Thought problems**		55.9 (7.7)	16.6	9.4 – 23.9	**0.0006**
**Internalizing problems**		54.0 (12.6)	18.6	11.0 – 26.1	**0.0096**
**Externalizing problems**		49.7 (11.5)	6.8	1.9 – 11.7	1.00
**Sluggish cognitive tempo**		57.2 (8.4)	24.5	16.1 – 32.8	**0.0006**

CI, confidence interval; CPT, Conners Continuous Performance Test-III; HRT, hit reaction time; ISI, inter-stimulus Intervals; SD, standard deviation.

*All neurocognitive and behavioral measures were transformed into age-adjusted T-scores (mean = 50; standard deviation [SD] = 10) using references provided by the test manuals. All T-scores were scaled such that a higher score was indicative of worse functioning or more severe problems.

^^^To estimate the prevalence of impairments within the study sample, impairment was defined as a score poorer than 1.5 standard deviation of age-adjusted T-scores of reference norms

^#^A one-sample t test was used to compare the survivors’ performance with population norms (T-score = 50). Boldface indicates statistical significance at P≦0.05. Statistical significance after applying Bonferroni correction.

Compared with the age- and sex-matched normative sample, survivors reported significantly more neurobehavioral symptoms in multiple domains (all *P* < 0.01, corrected for false discovery rate), with the exception of externalizing problems (**Table 2**). The highest impairment scores were reported for sluggish cognitive tempo (57.2 [8.4]; *P* = 0.0006). The prevalence of significant neurobehavioral deficits among survivors ranged from 6.8% to 24.5%.

### 3.3 Factors associated with attention outcomes

The distribution of survivors stratified by tertiles of the respective biomarkers are presented in **Supplement 4**. After adjustment for sex, age at evaluation, we found that survivors in the top tertile for MCP-1 reported higher impairment scores on impulsivity (CPT omissions: Est = 1.04, SE = 0.53; *P* = 0.050) and sustained attention (CPT hit reaction time SD: Est = 3.27, SE = 1.32; *P* = 0.015) (**Table 3**). Survivors with high IFN-γ and IL-13 levels showed more impairment on CPT detectability and CPT commission (all *P* < 0.05). No significant associations were observed for IL-8, MIP-1β, and TNF-α.

**Table 3:** Association between markers of inflammation and attention outcomes.

	CPT Detectability^			CPT Omissions^			CPT Commissions^			CPT Hit Reaction Time SD^		
Biological factors	Estimate	SE	*P*	Estimate	SE	*P*	Estimate	SE	*P*	Estimate	SE	*P*
IFN-γ												
Upper tertile	6.74	2.26	**0.0037**	0.81	0.64	0.21	7.31	2.37	**0.0027**	1.11	1.63	0.49
Other tertiles (referent)												
IL-8												
Upper tertile	1.62	2.18	0.45	0.26	0.59	0.66	2.89	2.27	0.20	-1.19	1.50	0.43
Other tertiles (referent)												
IL-13												
Upper tertile	5.10	2.27	**0.027**	0.36	0.63	0.56	7.33	2.34	**0.0023**	1.06	1.61	0.51
Other tertiles (referent)												
MCP-1												
Upper tertile	3.18	1.94	0.10	1.04	0.53	**0.050**	2.24	2.06	0.27	3.27	1.32	**0.015**
Other tertiles (referent)												
MIP-1b												
Upper tertile	-1.70	1.94	0.38	-0.27	0.53	0.60	-2.44	2.03	0.23	-0.37	1.34	0.78
Other tertiles (referent)												
TNF-α												
Upper tertile	2.79	2.01	0.16	0.24	0.55	0.66	2.20	2.13	0.30	0.18	1.40	0.89
Other tertiles (referent)												

IFN-γ, interferon gamma; IL, interleukin; MCP-1, monocyte chemoattractant protein-1; MIP-1β, macrophage inflammatory protein-1β; SE, standard error; TNF-α, tumor necrosis factor-α.

All statistical models were adjusted for gender, age at evaluation, age at diagnosis, and treatment factors. Boldface indicates statistical significance at P ≤ 0.05. Only measures on which the survivors differed from the normative samples at P < 0.05 (after Bonferroni correction, **Table 2**) were included in the analyses.

^A higher value was indicative of worse functioning

### 3.4 Factors associated with neurobehavioral outcomes

Survivors in the top tertile for IL-8 level had more self-reported thought problems (Est = 3.53, SE = 1.78; *P* = 0.050), and internalizing problems (Est = 6.52, SE = 2.91; *P* = 0.027) (**Table 4**). A higher TNF-α level was negatively correlated with internalizing problems (*P* = 0.0.35). No associations between plasma biomarkers and other neurobehavioral domains were identified.

**Table 4:** Association between markers of inflammation and neurobehavioral outcomes.

	Attention problems^			Thought problems^			Internalizing problems^			Sluggish cognitive tempo^		
Biological factors	Estimate	SE	*P*	Estimate	SE	*P*	Estimate	SE	*P*	Estimate	SE	*P*
IFN-γ												
Upper tertile	-0.72	1.95	0.71	-0.23	1.97	0.90	-1.28	3.23	0.69	0.083	2.10	0.96
Other tertiles (referent)												
IL-8												
Upper tertile	1.72	1.79	0.33	3.53	1.78	**0.050**	6.52	2.91	**0.027**	2.51	1.92	0.19
Other tertiles (referent)												
IL-13												
Upper tertile	-0.90	1.90	0.63	-0.24	1.92	0.89	1.30	3.15	0.67	0.076	2.05	0.97
Other tertiles (referent)												
MCP-1												
Upper tertile	0.011	1.62	0.99	0.54	1.64	0.73	3.33	2.67	0.21	2.08	1.74	0.23
Other tertiles (referent)												
MIP-1b												
Upper tertile	-1.01	1.60	0.52	-1.61	1.62	0.32	-1.74	2.66	0.51	-0.58	1.73	0.73
Other tertiles (referent)												
TNF-α												
Upper tertile	-1.77	1.66	0.29	-0.27	1.69	0.87	-5.79	2.72	**0.035**	1.04	1.80	0.56
Other tertiles (referent)												

IFN-γ, interferon gamma; IL, interleukin; MCP-1, monocyte chemoattractant protein-1; MIP-1β, macrophage inflammatory protein-1β; SE, standard error; TNF-α, tumor necrosis factor-α.

All statistical models were adjusted for gender, age at evaluation, age at diagnosis, and treatment factors. Boldface indicates statistical significance at P ≤ 0.05. Only measures on which the survivors differed from the normative samples at P < 0.05 (after Bonferroni correction, **Table 2**) were included in the analyses.

^A higher value was indicative of worse functioning

### 3.5 Clinical and treatment factors associated with biological markers

Survivors who had developed CHCs were more likely to have higher levels of IL-13 (RR = 4.58, 95% CI: 1.01–11.10) and TNF-α (RR = 1.44, 95% CI: 1.03–4.07) (**Table 5**). Survivors treated with the 20 g/m^2^ HDMTX regimen were more likely to have higher levels of IL-13 (RR = 5.22, 95% CI: 1.38–19.72) and MIP-1β (RR = 2.38, 95% CI: 1.32–12.28) than survivors who received the 8 g/m^2^ HDMTX regimen (**Table 5**). We did not identify any significant associations between biomarkers and other clinical factors.

**Table 5:** Association of clinical and treatment factors with markers of inflammation (exploratory analysis).

	IFN-γ		IL-8		IL-13		MCP-1		MIP-1β		TNF-α	
Clinical factors												
	RR	95% CI	RR	95% CI	RR	95% CI	RR	95% CI	RR	95% CI	RR	95% CI
Gender												
Male	0.77	0.28 – 2.10	0.82	0.32 – 2.09	1.10	0.41 – 2.92	1.37	0.58 – 3.20	0.90	0.38 – 2.09	1.23	0.51 – 2.97
Female (referent)												
**Age (years)**	0.77	0.28 – 2.10	0.82	0.32 – 2.09	1.10	0.41 – 2.92	1.37	0.58 – 3.20	0.90	0.38 – 2.09	1.23	0.51 – 2.97
**Time since diagnosis (years)**	1.02	0.95 – 1.09	1.00	0.93 – 1.07	1.06	0.98 – 1.14	0.97	0.91 – 1.03	1.01	0.95 – 1.07	0.98	0.92 – 1.04
CHCs												
Any	3.81	0.78 – 8.49	0.66	0.22 – 1.96	**4.58**	**1.01 – 11.10**	0.64	0.24 – 1.71	1.15	0.42 – 3.16	**1.44**	**1.03 – 4.07**
No (referent)												
Treatment factors												
Cranial radiation												
Yes	#		1.44	0.40 – 5.18	1.28	0.31 – 5.30	0.53	0.16 – 1.78	0.59	0.17 – 1.97	1.03	0.30 – 3.45
No (referent)												
HDMTX regimen												
20g/m^2^	#		1.69	0.50 – 5.64	**5.22**	**1.38 – 19.72**	0.61	0.22 – 1.69	**2.38**	**1.32 – 12.28**	2.21	0.72 – 6.80
8g/m^2^ (referent)												
No. of IT counts												
> 16 counts	1.00	0.35 – 2.85	1.55	0.55 – 4.38	**3.55**	**1.12 – 16.29**	1.45	0.58 – 3.59	0.77	0.30 – 1.94	1.25	0.49 – 3.18
≤ 16 counts (referent)												

CHC, chronic health condition; HDMTX, High-dose methotrexate; IFN-γ, interferon gamma; IL, interleukin; IT, intrathecal; MCP-1, monocyte chemoattractant protein-1; MIP-1β, macrophage inflammatory protein-1β; RR, risk ratio; TNF-α, tumor necrosis factor-α.

Boldface indicates statistical significance at P ≤ 0.05.

^#^Models for these variables were not constructed as the sample size for at least one of the cells had ≤5 counts.

### 3.6 Sensitivity analysis

The sensitivity analysis showed sex-specific association of biomarkers with functional outcomes. Male survivors performed worse than female survivors on multiple measures of attention (**Supplement 5**).

Higher levels of IFN-γ were significantly associated with worse attention outcomes (CPT detectability, omissions, and hit reaction time SD; all P < 0.05) in male survivors but not female survivors (**Supplement 6**). Female survivors with higher levels of IL-13 (P = 0.0019) and TNF-α (P = 0.0099) had higher impairment scores on CPT commissions.

Male survivors in the top tertile for IL-8 reported more thought problems than male survivors in other tertiles (*P* = 0.015) (**Supplement 7**). No sex-specific associations were observed for other biomarkers.

## 4 Discussion

This study explored the association of inflammation on attention and neurobehavioral outcomes among a cohort of young survivors of ALL. Higher systemic levels of IFN-γ and IL-13 were associated with inattentiveness, while IL-8 was positively correlated with internalizing neurobehavioral symptoms. Higher circulating IL-13 were found in survivors who developed CHCs, and in survivors who were treated with higher doses of HDMTX and intrathecal counts. Admittedly, we could not assess the persistence or chronicity of inflammation as this was a cross-sectional study. Nevertheless, our findings support the emerging evidence that a clinically significant link may exist between inflammation and cognitive and neurobehavioral deficits in cancer survivors, particularly in survivors with a compromised health status.

Higher levels of IL-13, IFN-γ, and MCP-1, but not TNF-α and IL-8, were found to be significantly associated with poorer attention outcomes in survivors. *In vivo* studies have suggested that IFN-γ can affect cognitive behaviors by reducing the viability of neural precursor cells and increasing cell death through caspase-3 expression (61), while MCP-1 can affect myelin degradation and neuronal loss through expression in microglia and macrophages (62). Both IFN-γ and MCP-1 have been reported to be elevated in patients with mild cognitive impairment in humans (62–64). However, the findings for IL-13 are controversial; some studies have reported that IL-13 can foster better cognitive outcomes by stimulating primary astrocytes to produce brain-derived neurotrophic factor (65), while other studies have shown that elevated IL-13 contributes to neuronal damage in patients with Alzheimer’s disease (66, 67). Interestingly, we found that survivors in the top tertile for IL-13 also had higher exposure to HDMTX and intrathecal injections. We acknowledge that this result is only suggestive of a potential association among previous anti-cancer treatment, cytokines, and attention and that the identified associations do not show causation. Furthermore, we only measured peripheral cytokines and might not reflect the actual inflammatory status in the brain. Nevertheless, substantial evidence from the literature has reported that cytokines can cross blood-brain barrier, and that cytokine-induced sickness behavior underlies the pathophysiology of many neuropsychiatric diseases (68, 69). Although the associations identified in our study are preliminary and modest in terms of effect size, such trend suggests inflammatory response induced by anti-cancer therapies on the clinical presentation of cognitive impairment in patients with cancer.

In the literature, TNF-α and IL-8 are found to be associated with cognitive performance in survivors of breast cancer (19, 50, 53); on the contrary, TNF-α and IL-8 were not associated with attention outcomes in our study. This might be because other studies have evaluated specific impairments in executive function and memory domains. Due to the constraints time and space constraint in the clinical setting of a public hospital, we could not conduct a full evaluation of other cognitive domains. Our findings need to be validated in a larger cohort of survivors with a more comprehensive neurocognitive battery.

Dysregulation of IL-8 levels has been observed in patients with neuropsychiatry symptoms (70–72). For example, a higher plasma IL-8 level was associated with symptoms of depression, trait negative affect, and perceived stress among healthy adults in the community (73). In our study, we found that survivors with higher IL-8 reported more thought problems and internalizing behavioral problems. A potential mechanism is that long-term survivors of childhood cancer experience significant chronic symptom burden, such as pain and fatigue (74), which may be a mediator between physiological stress and downstream functional outcomes. To illustrate this speculation, one study reported that among patients with breast cancer, emotional acceptance (as measured by the Acceptance of Emotion Scale) attenuates the relationship between IL-8 and TNF-α with cancer-related symptoms (75). Our future work includes evaluating the clustering effect of physiological markers of stress and cancer-related symptoms on cognitive and neurobehavioral outcomes in long-term survivors.

Within the pediatric oncology survivorship literature, there is emerging evidence that non–CNS-directed treatments can also induce neurotoxicity through CHCs in survivors (11, 12, 59). The exact mechanisms linking CHCs and cognitive outcomes have yet to be elucidated. In this analysis, we found higher levels of IL-13 and TNF-α in survivors who developed CHCs. Within the general population, higher inflammatory cytokines are found in patients with cardiovascular (76, 77), endocrine (77), and neurological (78) diseases. Stress and emotional dysregulation may also be precipitated by inflammatory cytokines that affect the hypothalamic–pituitary–adrenal axis responses in individuals with endocrine comorbidities (79, 80). Admittedly, our findings should be interpreted cautiously as the small sample size did not allow us to conduct meaningful analyses on the associations between inflammatory makers and specific CHCs. The pathophysiology and mechanisms behind each CHC are different. Future work should focus on examining the mediatory role of inflammatory markers between specific CHC development and its impact on cognitive and neurobehavioral deficits in survivors.

There is now growing evidence to support sex-specific differences in the pathophysiology underlying cognitive and neurobehavioral outcomes among survivors (17, 21, 22, 59, 60). For example, one study found that male survivors of ALL with higher TNF-α and oxidized low-density lipoprotein displayed more neurobehavioral problems with initiation and organization skills; but such associations were not observed in female survivors (17). Our study found that higher levels of IFN-γ and IL-8 were significantly associated with worse attention and neurobehavioral outcomes, respectively, in male survivors but not female survivors. One plausible explanation is that inhibitory and emotional control involves “higher-order cognitive skills,” which are typically considered to be dependent upon prefrontal brain areas and gray matter integrity. Therefore, difficulties on tasks mediated by gray matter might be reflected in males as some studies have suggested that the rate of gray matter development in boys during adolescence is slower than that in girls (21, 81). Admittedly, our sex-stratified results should be interpreted cautiously due to the small sample size. Future efforts should focus on investigating the relationship between specific inflammatory biomarkers and functional outcomes in survivors and on evaluating the moderating effect of sex and risk after brain insult.

Our results should be interpreted in the context of the following limitations. Our study sample size was relatively small and did not include an age-matched healthy comparison control group. However, both the attention and neurobehavioral outcomes are scored according to population norms. Although we could not compare the inflammatory status between survivors and non-cancer controls, eliciting inflammatory responses is a well-established mechanism of conventional cancer treatments, and such effects that sustained long after cancer treatment may lead to chronic low-grade systemic inflammation in survivors (82, 83). Our findings should be validated prospectively in larger cohorts and compared with an age- and matched control group. Other factors that could potentially affect inflammatory status and neurobehavioral outcomes were not evaluated in detail due to the constraints of resources and time within a clinical setting. For example, physical activity, diet, and socio-environmental factors may affect the associations of interest. On-treatment neurotoxicity and neurological complications, such as acute leukoencephalopathy and persistent peripheral neuropathy, might also affect long-term neurocognitive function (84, 85). Future work should also investigate the association of treatment and chronic inflammatory status on long-term neurological outcomes using contemporary and sensitive neurophysiological techniques, such as auditory evoked potential P300 analysis, and its correlation with neurobehavioral outcomes (86). Finally, there is large inter- and intra-patient variability in inflammatory status, as evidenced by the wide variance in cytokine levels among survivors. Even though we accounted for age and treatment factors in our multivariable models, we cannot eliminate the potential confounding effect of aging and subclinical cancer progression. Future prospective studies may strengthen this evidence base by establishing the temporal relationships between inflammation and functional outcomes through longitudinal assessments.

The aforementioned limitations notwithstanding, this mechanistic work may contribute to important clinical implications in the design of interventions to mitigate cognitive decline. For example, there is emerging evidence to support behavioral interventions for improving health and psychosocial outcomes in cancer survivors (87–89). Our study and collective findings from the literature may provide support on the application of markers of inflammation to assess or monitor the effectiveness of pharmacological and non-pharmacological interventions, particularly behavioral interventions. For example, a recent study reported that childhood cancer survivors who received a physical exercise intervention had significantly lower systemic-immune-inflammation index than survivors in the control group; this study demonstrated the potential anti-inflammatory effects of exercise on cancer survivors (90). Such an application would be in line with the mission of the United States National Institute of Health Science of Behavior Change Program (91): to make behavior change research more impactful, targeted, and systematic by promoting a mechanism-focused, experimental medicine approach.

## 5 Conclusions

Our findings suggested that systemic inflammation may be one of the critical pathophysiological basis driving attention impairment and neurobehavioral symptoms in long-term survivors of pediatric ALL. Male survivors appear to be more susceptible to the cognitive effects of systemic inflammation. Future research with this cohort should include prospective follow-up and a more comprehensive assessment of neurocognitive function as well as neuroimaging in order to capture concurrent measurements of white-matter development and performance. A systematic approach to evaluating the moderating effect of sex, treatment exposures, and health status trajectories during survivorship should also be pursued so that more targeted interventions may be tailored to high-risk subgroups.

## Data availability statement

The datasets presented in this article are not readily available due to patient privacy and confidentiality. Requests to access the datasets should be directed to the corresponding author.

## Ethics statement

The studies involving human participants were reviewed and approved by the Joint Chinese University of Hong Kong – New Territories East Cluster Clinical Research Ethics Committee (Ref: 2017.701). The patients/participants provided their written informed consent to participate in this study.

## Author contributions

Concept and design: YC, CKL, CL, AC, KT. Literature review: YC, RH. Acquisition of data: YC, CKL, CL. Assay of biological samples: KT. Analysis of data: YC. Date interpretation: All authors. Drafting of the manuscript: YC. Critical revision for important intellectual content: All authors. All authors contributed to the article and approved the submitted version.
