# Supplementary material for: Association of markers of inflammation on attention and neurobehavioral outcomes in survivors of childhood acute lymphoblastic leukemia
Source: Front Oncol. 2023 Jun 21;13:1117096. doi: 10.3389/fonc.2023.1117096 (PMC10320851; doi:10.3389/fonc.2023.1117096)
Supplement: Supplementary file 1 [file Table_1.docx]

Supplement 1: Evidence from Selected Literature Supporting the Association between the Cytokines of Interest and Cognitive Impairment

| Cytokines of Interest | Supporting evidence from the literature | References |
| --- | --- | --- |
| TNF-α | - High level of TNF-α associated with worse executive function in male survivors of childhood ALL | (Cheung et al., 2017) (Study) |
|  | - High level of TNF-α associated with reduced left hippocampal volumes and cognitive performance in patients with breast cancer | (Kesler et al., 2013) (Study) |
|  | - Longitudinal decline in sTNF-RII significantly correlated with fewer memory complaints in patients with breast cancer | (Ganz et al., 2013) (Study) |
|  | - TNF-α signaling exacerbates both Aβ and tau pathologies in patients with Alzheimer’s disease - TNF-α plasma levels observed in patients with MCI as compared to cognitively normal individuals | (Decourt et al., 2017) (Review) |
|  | - Weak to moderate correlations observed between TNF-α levels and different degrees of cognitive impairment in studies of patients with cancer | (Cheung et al., 2013) (Review) |
|  | - A significant elevation of TNF-α was found in the serum of patients who displayed rapidly progressive form of AD. | (Stoeck et al., 2014) (Study) |
|  | Higher levels of TNF-α associated with better cognitive processing performance in breast cancer patients receiving chemotherapy | (Williams et al., 2018) (Study) |
| IL-8 | - Higher serum IL-8 concentrations in AD patients as compared with healthy controls. | (Su et al., 2019) (Review and meta-analysis) |
|  | - IL-8 levels were negatively associated with executive functioning in early-stage breast cancer survivors at one-year after the initiation of chemotherapy. | (Lyon et al., 2016) (Study) |
|  | - Plasma IL-8 levels were elevated among early-stage breast cancer survivors who reported persistent cognitive problems. | (Toh et al., 2020) (Study) |
|  | - Higher plasma IL-8 levels were associated with worse processing speed and attention in breast cancer patients before and after chemotherapy. | (Belcher et al., 2022) (Study) |
|  | - Higher serum IL-8 level was correlated with white matter hyperintensities in patients with AD and in individuals who were cognitively impaired but no dementia. | (Zhu et al., 2017) (Study) |
|  | - Plasma IL-8 levels were negatively associated with executive function in children with sickle cell disease. | (Andreotti et al., 2015) (Study) |
| IL-13 | - Higher plasma IL-13 were associated with lower perceived cognitive abilities in pre-chemotherapy breast cancer group and in healthy control. - Higher IL-13 were associated with poorer verbal fluency in the post-chemotherapy group. | (Chen et al., 2021) (Study) |
|  | - Plasma IL-13 levels were negatively associated with executive function in children with sickle cell disease. | (Andreotti et al., 2015) (Study) |
|  | - A significant elevation of IL-13 was found in the serum of patients who displayed rapidly progressive form of AD. | (Stoeck et al., 2014) (Study) |
|  | - Higher plasma IL-13 levels associated with poorer memory and executive function in healthy older adults. | (Serre-Miranda et al., 2020) (Study) |
| IFN-γ | - IFN-γ levels were negatively associated with executive functioning in early-stage breast cancer survivors at one-year after the initiation of chemotherapy. | (Lyon et al., 2016) (Study) |
|  | - Higher plasma IFN-γ levels were predictive of slower cognitive decline independent of amyloid beta among cognitively unimpaired older adults. | (Yang et al., 2022) (study) |
|  | - Among older adults who were cognitively impaired but no dementia, those with genetic variants that produce higher expression of INF-γ showed better normalized cognitive performance. | (Fraga et al., 2017) (study) |
| MCP-1 | - Higher level of peripheral MCP-1 in MCI patients as compared to individuals with normal cognitive function. | (Shen et al., 2019) (Meta-analysis) |
|  | - MCP-1 levels were negatively correlated with psychomotor speed at one year after the initiation of chemotherapy in early-stage breast cancer patients. - MCP-1 levels were negatively correlated with psychomotor speed and complex attention at two years after the initiation of chemotherapy | (Lyon et al., 2016) (Study) |
|  | - Higher levels of serum MCP-1 significantly associated with better executive function and visual memory among breast cancer patients receiving chemotherapy. | (Williams et al., 2018) (Study) |
|  | - Increase in MCP-1 level was associated with worse executive function and verbal fluency in from pre- to post-chemotherapy in breast cancer patients. | (Janelsins et al., 2022) (Study) |
|  | - MCP-1 levels in the cerebrospinal fluid significantly increased in patients with amyotrophic lateral sclerosis, as compared with controls. | (Chen et al., 2018) (Meta-analysis) |
| MIP-1β | - Psychomotor speed was negatively associated with MIP-1β at one year after the initiation of chemotherapy in early-stage breast cancer patients. | (Lyon et al., 2016) (Study) |
|  | - Lower fibrillary amyloid aggregates levels associated with higher cerebrospinal fluid MIP-1β levels in patients with cognitive complaints. | (Aksnes et al., 2021) (study) |

AD: Alzheimer’s Disease; IFN*-*γ*:* interferon gamma; IL: interleukin; MCI: mild cognitive impairment; MCP-1: monocyte chemoattractant protein*-*1; MIP-1β: macrophage inflammatory protein-1β; TNF-α: tumor necrosis factor-α

Aksnes, M., Aass, H. C. D., Tiiman, A., Edwin, T. H., Terenius, L., Bogdanović, N., . . . Knapskog, A.-B. (2021). Associations of cerebrospinal fluid amyloidogenic nanoplaques with cytokines in Alzheimer's disease. *Translational neurodegeneration*, *10*(1), 18-18. <https://doi.org/10.1186/s40035-021-00244-3>

Andreotti, C., King, A. A., Macy, E., Compas, B. E., & DeBaun, M. R. (2015). The Association of Cytokine Levels With Cognitive Function in Children With Sickle Cell Disease and Normal MRI Studies of the Brain. *Journal of child neurology*, *30*(10), 1349-1353. <https://doi.org/10.1177/0883073814563140>

Belcher, E. K., Culakova, E., Gilmore, N. J., Hardy, S. J., Kleckner, A. S., Kleckner, I. R., . . . Janelsins, M. C. (2022). Inflammation, Attention, and Processing Speed in Patients With Breast Cancer Before and After Chemotherapy. *J Natl Cancer Inst*, *114*(5), 712-721. <https://doi.org/10.1093/jnci/djac022>

Chen, V. C.-H., Lin, C.-K., Hsiao, H.-P., Tzang, B.-S., Hsu, Y.-H., Wu, S.-I., & Stewart, R. (2021). Effects of Cancer, Chemotherapy, and Cytokines on Subjective and Objective Cognitive Functioning Among Patients with Breast Cancer. *Cancers*, *13*(11), 2576. <https://doi.org/10.3390/cancers13112576>

Chen, X., Hu, Y., Cao, Z., Liu, Q., & Cheng, Y. (2018). Cerebrospinal Fluid Inflammatory Cytokine Aberrations in Alzheimer's Disease, Parkinson's Disease and Amyotrophic Lateral Sclerosis: A Systematic Review and Meta-Analysis. *Front Immunol*, *9*, 2122-2122. <https://doi.org/10.3389/fimmu.2018.02122>

Cheung, Y. T., Brinkman, T. M., Mulrooney, D. A., Mzayek, Y., Liu, W., Banerjee, P., . . . Krull, K. R. (2017). Impact of sleep, fatigue, and systemic inflammation on neurocognitive and behavioral outcomes in long-term survivors of childhood acute lymphoblastic leukemia. *Cancer*, *123*(17), 3410-3419. <https://doi.org/10.1002/cncr.30742> [doi]

Cheung, Y. T., Lim, S. R., Ho, H. K., & Chan, A. (2013). Cytokines as mediators of chemotherapy-associated cognitive changes: current evidence, limitations and directions for future research. *PloS one*, *8*(12), e81234-e81234. <https://doi.org/10.1371/journal.pone.0081234>

Decourt, B., Lahiri, D. K., & Sabbagh, M. N. (2017). Targeting Tumor Necrosis Factor Alpha for Alzheimer's Disease. *Current Alzheimer research*, *14*(4), 412-425. <https://doi.org/10.2174/1567205013666160930110551>

Fraga, V. G., Guimarães, H. C., Teixeira, A. L., Barbosa, M. T., Carvalho, M. G., Caramelli, P., & Gomes, K. B. (2017). Polymorphisms in cytokine genes influence cognitive and functional performance in a population aged 75 years and above. *Int J Geriatr Psychiatry*, *32*(12), 1401-1410. <https://doi.org/10.1002/gps.4627>

Ganz, P. A., Bower, J. E., Kwan, L., Castellon, S. A., Silverman, D. H., Geist, C., . . . Cole, S. W. (2013). Does tumor necrosis factor-alpha (TNF-α) play a role in post-chemotherapy cerebral dysfunction? *Brain, behavior, and immunity*, *30 Suppl*(Suppl), S99-108. <https://doi.org/10.1016/j.bbi.2012.07.015>

Janelsins, M. C., Lei, L., Netherby-Winslow, C., Kleckner, A. S., Kerns, S., Gilmore, N., . . . Culakova, E. (2022). Relationships between cytokines and cognitive function from pre- to post-chemotherapy in patients with breast cancer. *Journal of neuroimmunology*, *362*, 577769. <https://doi.org/https://doi.org/10.1016/j.jneuroim.2021.577769>

Kesler, S., Janelsins, M., Koovakkattu, D., Palesh, O., Mustian, K., Morrow, G., & Dhabhar, F. S. (2013). Reduced hippocampal volume and verbal memory performance associated with interleukin-6 and tumor necrosis factor-alpha levels in chemotherapy-treated breast cancer survivors. *Brain, behavior, and immunity*, *30 Suppl*(0), S109-S116. <https://doi.org/10.1016/j.bbi.2012.05.017>

Lyon, D. E., Cohen, R., Chen, H., Kelly, D. L., McCain, N. L., Starkweather, A., . . . Jackson-Cook, C. K. (2016). Relationship of systemic cytokine concentrations to cognitive function over two years in women with early stage breast cancer. *Journal of neuroimmunology*, *301*, 74-82. <https://doi.org/10.1016/j.jneuroim.2016.11.002>

Serre-Miranda, C., Roque, S., Santos, N. C., Costa, P., Sousa, N., Palha, J. A., & Correia-Neves, M. (2020). Cognition Is Associated With Peripheral Immune Molecules in Healthy Older Adults: A Cross-Sectional Study [Original Research]. *Front Immunol*, *11*. <https://doi.org/10.3389/fimmu.2020.02045>

Shen, X.-N., Niu, L.-D., Wang, Y.-J., Cao, X.-P., Liu, Q., Tan, L., . . . Yu, J.-T. (2019). Inflammatory markers in Alzheimer’s disease and mild cognitive impairment: a meta-analysis and systematic review of 170 studies. *J Neurol Neurosurg Psychiatry*, *90*(5), 590-598. <https://doi.org/10.1136/jnnp-2018-319148>

Stoeck, K., Schmitz, M., Ebert, E., Schmidt, C., & Zerr, I. (2014). Immune responses in rapidly progressive dementia: a comparative study of neuroinflammatory markers in Creutzfeldt-Jakob disease, Alzheimer's disease and multiple sclerosis. *J Neuroinflammation*, *11*(1), 170. <https://doi.org/10.1186/s12974-014-0170-y>

Su, C., Zhao, K., Xia, H., & Xu, Y. (2019). Peripheral inflammatory biomarkers in Alzheimer's disease and mild cognitive impairment: a systematic review and meta-analysis. *Psychogeriatrics*, *19*(4), 300-309. <https://doi.org/10.1111/psyg.12403>

Toh, Y. L., Wang, C., Ho, H. K., & Chan, A. (2020). Distinct cytokine profiles across trajectories of self-perceived cognitive impairment among early-stage breast cancer survivors. *Journal of neuroimmunology*, *342*, 577196. <https://doi.org/https://doi.org/10.1016/j.jneuroim.2020.577196>

Williams, A. M., Shah, R., Shayne, M., Huston, A. J., Krebs, M., Murray, N., . . . Janelsins, M. C. (2018). Associations between inflammatory markers and cognitive function in breast cancer patients receiving chemotherapy. *Journal of neuroimmunology*, *314*, 17-23. <https://doi.org/https://doi.org/10.1016/j.jneuroim.2017.10.005>

Yang, H. S., Zhang, C., Carlyle, B. C., Zhen, S. Y., Trombetta, B. A., Schultz, A. P., . . . Tanzi, R. E. (2022). Plasma IL-12/IFN-γ axis predicts cognitive trajectories in cognitively unimpaired older adults. *Alzheimers Dement*, *18*(4), 645-653. <https://doi.org/10.1002/alz.12399>

Zhu, Y., Chai, Y. L., Hilal, S., Ikram, M. K., Venketasubramanian, N., Wong, B.-S., . . . Lai, M. K. P. (2017). Serum IL-8 is a marker of white-matter hyperintensities in patients with Alzheimer's disease. *Alzheimers Dement (Amst)*, *7*, 41-47. <https://doi.org/https://doi.org/10.1016/j.dadm.2017.01.001>

Supplement 2: Flow Diagram

**Screened**

(n=160)

**Eligible**

(n=143)

**Excluded (n=17)**

Relapsed (n=13)

Pre-existing cognitive impairment (n=3)

Secondary malignancy (n=1)

Non-participants (n=17)

Contact not successful (default appointment [5], uncontactable [4]) (n=9)

Refusal (did not have time [5], not interested [1]) (n=6)

Withdrawal (did not have time to finish) (n=2)

**Completed all assessments**

(n=126)

Excluded from the current analysis (n=24)

Did not provide blood samples (n=12)

Unmeasurable samples (n = 6)

Incomplete diagnosis information or treatment protocol information (n=6)

**Analyzed**

(n=102)

Supplement 3: Specific Chronic Health Conditions Diagnoses

| Chronic health conditions | Diagnoses (ICD-9 code) |
| --- | --- |
| Cardiopulmonary | Essential hypertension (401)  Acute myocardial infarction (410)  Acute pulmonary heart disease (415) |
| Endocrine/metabolic | Hypothyroidism (244)  Secondary diabetes mellitus (249)  Disorders of parathyroid gland (252)  Ovarian dysfunction (256)  Testicular dysfunction (257)  Disorders of lipid metabolism (272) |
| Neurology | Epilepsy (345)  Migraine (346)  Peripheral neuropathy (356) |

Treatment-related chronic health conditions were defined as health conditions that were diagnosed during or after the completion of cancer treatment. Conditions that existed before cancer diagnosis and treatment were not considered in the analysis.

Supplement 4: Levels of Biomarkers Stratified by Tertiles

|  | Other tertiles | | | Top tertile | | |
| --- | --- | --- | --- | --- | --- | --- |
| Biological marker | *n* | Median (IQR) | Range | *n* | Median (IQR) | Range |
| IFN-γ (pg/ml) | 82 | 0.025 (0.025 - 0.025) | 0.025 - 0.025 | 20 | 0.32 (0.2 - 0.7) | 0.05 - 2.8 |
| IL-8 (pg/ml) | 79 | 0.01 (0.01 - 0.01) | 0.01 - 0.01 | 23 | 0.73 (0.4 - 1.9) | 0.2 - 2.6 |
| IL-13 (pg/ml) | 79 | 0.025 (0.025 – 0.025) | 0.025 – 0.025 | 23 | 0.28 (0.08 – 0.4) | 0.05 – 6.0 |
| MCP-1 (pg/ml) | 69 | 30.2 (24.3 – 30.0) | 6.4 – 38.7 | 33 | 44.6 (40.8 – 51.1) | 39.3 - 65.9 |
| MIP-1β (pg/ml) | 69 | 12.3 (8.8 - 15.4) | 2.6 - 18.4 | 33 | 24.0 (22.1 – 26.9) | 19.2 – 43.0 |
| TNF-α (pg/ml) | 73 | 2.3 (0.6 - 5.2) | 0.6 - 7.7 | 29 | 10.6 (8.7 – 11.3) | 7.8 - 363.8 |

IFN*-*γ*:* interferon gamma; IL: interleukin; MCP-1: monocyte chemoattractant protein*-*1*;* MIP-1β: macrophage inflammatory protein-1β; TNF-α: tumor necrosis factor-*α*

Supplement 5: Attention and Behavioral Outcomes, Stratified by Gender

|  |  | Neurocognitive outcomes | | | |
| --- | --- | --- | --- | --- | --- |
|  | | | **Mean (SD)**  *T*-Scores* | **Mean (SD)**  *T*-Scores* | ***P***# |
| Attention | | | **Female** | **Male** |  |
| CPT HRT ISI change (sustained attention) | | | 49.8 (7.7) | 52.0 (7.4) | 0.19 |
| CPT HRT SD (sustained attention) | | | 54.5 (6.8) | 57.5 (5.8) | **0.034** |
| CPT Variability (inattentiveness) | | | 52.3 (9.8) | 51.9 (9.7) | 0.81 |
| CPT Omissions (inattentiveness) | | | 53.4 (2.8) | 55.3 (2.2) | **<.0001** |
| CPT Detectability (inattentiveness) | | | 54.0 (9.1) | 56.1 (9.1) | 0.28 |
| CPT Perseverations (impulsivity) | | | 50.1 (6.8) | 52.8 (7.2) | **<.0001** |
| CPT Commissions (impulsivity) | | | 53.4 (7.2) | 55.3 (8.0) | 0.10 |
|  |  | **Behavioral outcomes (Syndrome scales)** | | | |
|  | | | **Mean (SD)**  *T*-Scores* | **Impaired %**^ | ***P***# |
| Attention problems | | | 55.9 (7.3) | 55.4 (8.3) | 0.42 |
| Thought problems | | | 56.0 (7.4) | 55.9 (8.0) | 0.91 |
| Internalizing problems | | | 53.4 (12.1) | 54.5 (13.0) | 0.70 |
| Externalizing problems | | | 49.8 (11.3) | 49.7 (11.8) | 0.86 |
| Sluggish cognitive tempo | | | 56.7 (7.8) | 57.5 (8.8) | 0.76 |

CI: confidence interval; CPT: Conners Continuous Performance Test-III; HRT: hit reaction time; ISI: inter-stimulus Intervals; SD: standard deviation

* All neurocognitive and behavioral measures were transformed into *T*-scores (mean = 50; standard deviation [SD] = 10) using references provided by the test manuals. All *T*-scores were scaled such that a higher score was indicative of worse functioning or more severe problems.

# The Mann-Whitney U test was used to compare the male versus female survivors’ performance. Boldface indicates statistical significance at *P*≦0.05.

Supplement 6: Association between Markers of Inflammation and Attention Outcomes, Stratified by Gender

|  |  | CPT Detectability^ | | | CPT Omissions^ | | | CPT Commissions^ | | | CPT Hit Reaction Time SD^ | | |
| --- | --- | --- | --- | --- | --- | --- | --- | --- | --- | --- | --- | --- | --- |
|  |  | Est | SE | *P* | Est | SE | *P* | Est | SE | *P* | Est | SE | *P* |
| Biological factors | | | | | | | | | | | | | |
|  | IFN-γ |  |  |  |  |  |  |  |  |  |  |  |  |
| Male | Upper tertile (n=12) | 9.34 | 2.78 | **0.0015** | 2.22 | 0.69 | **0.0024** | 6.75 | 3.04 | **0.030** | 4.60 | 1.91 | **0.020** |
|  | Other tertiles (ref) (n=45) | - | - | - | - | - | - | - | - | - | - | - | - |
| Female | Upper tertile (n=8) | 2.83 | 3.78 | 0.45 | -1.18 | 1.15 | 0.30 | 7.69 | 3.85 | 0.052 | -4.14 | 2.60 | 0.11 |
|  | Other tertiles (ref) (n=37) | - | - | - | - | - | - | - | - | - | - | - | - |
|  | IL-8 |  |  |  |  |  |  |  |  |  |  |  |  |
| Male | Upper tertile (n=12) | 3.51 | 2.95 | 0.24 | 1.03 | 0.72 | 0.16 | 5.14 | 3.02 | 0.094 | 0.96 | 1.96 | 0.62 |
|  | Other tertiles (ref) (n=45) | - | - | - | - | - | - | - | - | - | - | - | - |
| Female | Upper tertile (n=11) | 0.09 | 3.31 | 0.97 | -0.58 | 1.01 | 0.56 | 0.82 | 3.51 | 0.81 | -3.11 | 2.28 | 0.17 |
|  | Other tertiles (ref) (n=34) | - | - | - | - | - | - | - | - | - | - | - | - |
|  | IL-13 |  |  |  |  |  |  |  |  |  |  |  |  |
| Male | Upper tertile (n=12) | 4.74 | 3.19 | 0.14 | 1.38 | 0.78 | 0.083 | 4.10 | 3.32 | 0.22 | 1.70 | 2.12 | 0.42 |
|  | Other tertiles (ref) (n=45) | - | - | - | - | - | - | - | - | - | - | - | - |
| Female | Upper tertile (n=11) | 5.82 | 3.35 | 0.090 | -0.69 | 1.06 | 0.51 | 10.87 | 3.26 | **0.0019** | 0.98 | 2.44 | 0.69 |
|  | Other tertiles (ref) (n=34) | - | - | - | - | - | - | - | - | - | - | - | - |
|  | MCP-1 |  |  |  |  |  |  |  |  |  |  |  |  |
| Male | Upper tertile (n=20) | 4.85 | 2.51 | 0.059 | 0.95 | 0.63 | 0.13 | 3.61 | 2.65 | 0.17 | 5.03 | 1.56 | **0.0022** |
|  | Other tertiles (ref) (n=37) | - | - | - | - | - | - | - | - | - | - | - | - |
| Female | Upper tertile (n=13) | -0.67 | 3.21 | 0.83 | -1.22 | 0.96 | 0.21 | -0.077 | 3.41 | 0.98 | -0.012 | 2.26 | 0.99 |
|  | Other tertiles (ref) (n=32) | - | - | - | - | - | - | - | - | - | - | - | - |
|  | MIP-1b |  |  |  |  |  |  |  |  |  |  |  |  |
| Male | Upper tertile (n=18) | -1.62 | 2.60 | 0.53 | -0.35 | 0.64 | 0.58 | -1.29 | 2.70 | 0.63 | -0.52 | 1.71 | 0.75 |
|  | Other tertiles (ref) (n=39) | - | - | - | - | - | - | - | - | - | - | - | - |
| Female | Upper tertile (n=15) | -1.39 | 3.03 | 0.64 | -0.18 | 0.93 | 0.84 | -3.66 | 3.17 | 0.25 | 0.26 | 2.14 | 0.90 |
|  | Other tertiles (ref) (n=30) | - | - | - | - | - | - | - | - | - | - | - | - |
|  | TNF-α |  |  |  |  |  |  |  |  |  |  |  |  |
| Male | Upper tertile (n=17) | -0.81 | 2.64 | 0.75 | -0.21 | 0.65 | 0.74 | -2.73 | 2.72 | 0.31 | -1.03 | 1.73 | 0.55 |
|  | Other tertiles (ref) (n=40) | - | - | - | - | - | - | - | - | - | - | - | - |
| Female | Upper tertile (n=12) | 7.73 | 3.07 | **0.016** | 0.88 | 1.00 | 0.38 | 8.73 | 3.22 | **0.0099** | 1.49 | 2.31 | 0.52 |
|  | Other tertiles (ref) (n=33) | - | - | - | - | - | - | - | - | - | - | - | - |

Est: estimate; IFN*-*γ*:* interferon gamma; IL: interleukin; MCP-1: monocyte chemoattractant protein*-*1*;* MIP-1β: macrophage inflammatory protein-1β; SE: standard error; TNF-α: tumor necrosis factor-*α*

All statistical models were adjusted for gender, age at evaluation, age at diagnosis, and treatment factors. Boldface indicates statistical significance at *P*≤0.05.

^ A higher value was indicative of worse functioning

Supplement 7: Association between Markers of Inflammation and Behavioral Outcomes, Stratified by Gender

|  |  | Attention problems^ | | | Thought problems^ | | | Internalizing problems^ | | | Sluggish cognitive tempo^ | | |
| --- | --- | --- | --- | --- | --- | --- | --- | --- | --- | --- | --- | --- | --- |
|  |  | Est | SE | *P* | Est | SE | *P* | Est | SE | *P* | Est | SE | *P* |
| Biological factors | |  |  |  |  |  |  |  |  |  |  |  |  |
|  | IFN-γ |  |  |  |  |  |  |  |  |  |  |  |  |
| Male | Upper tertile (n=12) | -3.32 | 2.51 | 0.19 | -1.77 | 2.63 | 0.50 | -5.10 | 4.30 | 0.24 | -2.90 | 2.84 | 0.31 |
|  | Other tertiles (ref) (n=45) | - | - | - | - | - | - | - | - | - | - | - | - |
| Female | Upper tertile (n=8) | 2.30 | 2.91 | 0.43 | 1.42 | 2.85 | 0.62 | 4.04 | 4.96 | 0.42 | 3.90 | 3.14 | 0.22 |
|  | Other tertiles (ref) (n=37) | - | - | - | - | - | - | - | - | - | - | - | - |
|  | IL-8 |  |  |  |  |  |  |  |  |  |  |  |  |
| Male | Upper tertile (n=12) | 2.09 | 2.46 | 0.39 | 6.11 | 2.43 | **0.015** | 6.17 | 4.15 | 0.14 | 3.29 | 2.74 | 0.23 |
|  | Other tertiles (ref) (n=45) | - | - | - | - | - | - | - | - | - | - | - | - |
| Female | Upper tertile (n=11) | 1.89 | 2.53 | 0.45 | 1.41 | 2.47 | 0.57 | 7.64 | 4.18 | 0.074 | 1.85 | 2.77 | 0.50 |
|  | Other tertiles (ref) (n=34) | - | - | - | - | - | - | - | - | - | - | - | - |
|  | IL-13 |  |  |  |  |  |  |  |  |  |  |  |  |
| Male | Upper tertile (n=12) | -2.23 | 2.62 | 0.39 | -2.62 | 2.71 | 0.33 | 1.11 | 4.51 | 0.80 | -2.83 | 2.93 | 0.34 |
|  | Other tertiles (ref) (n=45) | - | - | - | - | - | - | - | - | - | - | - | - |
| Female | Upper tertile (n=11) | 0.99 | 2.67 | 0.71 | 2.90 | 2.57 | 0.26 | 1.87 | 4.56 | 0.68 | 3.47 | 2.87 | 0.23 |
|  | Other tertiles (ref) (n=34) | - | - | - | - | - | - | - | - | - | - | - | - |
|  | MCP-1 |  |  |  |  |  |  |  |  |  |  |  |  |
| Male | Upper tertile (n=20) | -0.59 | 2.15 | 0.78 | -0.44 | 2.24 | 0.84 | 2.07 | 3.68 | 0.57 | 0.70 | 2.42 | 0.77 |
|  | Other tertiles (ref) (n=37) | - | - | - | - | - | - | - | - | - | - | - | - |
| Female | Upper tertile (n=13) | 1.64 | 2.46 | 0.50 | 2.52 | 2.38 | 0.29 | 5.03 | 4.15 | 0.23 | 4.83 | 2.59 | 0.070 |
|  | Other tertiles (ref) (n=32) | - | - | - | - | - | - | - | - | - | - | - | - |
|  | MIP-1b |  |  |  |  |  |  |  |  |  |  |  |  |
| Male | Upper tertile (n=18) | -1.59 | 2.15 | 0.46 | -0.95 | 2.24 | 0.67 | -1.78 | 3.69 | 0.63 | -2.16 | 2.41 | 0.37 |
|  | Other tertiles (ref) (n=39) | - | - | - | - | - | - | - | - | - | - | - | - |
| Female | Upper tertile (n=15) | 0.09 | 2.34 | 0.96 | -1.89 | 2.26 | 0.40 | -1.21 | 3.99 | 0.76 | 1.49 | 2.55 | 0.56 |
|  | Other tertiles (ref) (n=30) | - | - | - | - | - | - | - | - | - | - | - | - |
|  | TNF-α |  |  |  |  |  |  |  |  |  |  |  |  |
| Male | Upper tertile (n=17) | -0.74 | 2.19 | 0.73 | 0.064 | 2.27 | 0.97 | -4.52 | 3.69 | 0.22 | 1.03 | 2.46 | 0.67 |
|  | Other tertiles (ref) (n=40) | - | - | - | - | - | - | - | - | - | - | - | - |
| Female | Upper tertile (n=12) | -4.10 | 2.46 | 0.10 | -1.33 | 2.47 | 0.59 | -7.67 | 4.17 | 0.073 | 0.51 | 2.78 | 0.85 |
|  | Other tertiles (ref) (n=33) | - | - | - | - | - | - | - | - | - | - | - | - |

Est: estimate; IFN*-*γ*:* interferon gamma; IL: interleukin; MCP-1: monocyte chemoattractant protein*-*1*;* MIP-1β: macrophage inflammatory protein-1β; SE: standard error; TNF-α: tumor necrosis factor-*α*

All statistical models were adjusted for gender, age at evaluation, age at diagnosis, and treatment factors. Boldface indicates statistical significance at *P*≤0.05.

^ A higher value was indicative of worse functioning
